# *VEGF* and *bFGF* Gene Polymorphisms in Patients with Non-Hodgkin's Lymphoma

**DOI:** 10.1155/2013/159813

**Published:** 2013-08-26

**Authors:** Tomasz Wróbel, Grzegorz Mazur, Justyna Dzietczenia, Katarzyna Gębura, Kazimierz Kuliczkowski, Katarzyna Bogunia-Kubik

**Affiliations:** ^1^Department of Haematology, Blood Neoplasms and Bone Marrow Transplantation, Wroclaw Medical University, L. Pasteura 4, 50-367 Wroclaw, Poland; ^2^Department of Internal, Occupational Diseases and Hypertension, Wroclaw Medical University, Borowska 213, 50-556 Wroclaw, Poland; ^3^Laboratory of Clinical Immunogenetics and Pharmacogenetics, L. Hirszfeld Institute of Immunology and Experimental Therapy, Polish Academy of Sciences, R. Weigla 12, 53-114 Wroclaw, Poland

## Abstract

Angiogenesis and lymphangiogenesis are important in the proliferation and survival of the malignant hematopoietic neoplasms, including non-Hodgkin's lymphomas (NHLs). Vascular endothelial growth factor (VEGF) and basic fibroblast growth factor (bFGF) play an important role in the initiation of angiogenesis. Both VEGF and bFGF have been reported to have prognostic significance in NHL. The present study aimed to determine an association between the *VEGF* and *bFGF* gene polymorphisms and disease susceptibility and progression. *VEGF* (rs3025039; 936 C>T) and *bFGF* (rs308395, −921 G>C) variants were determined in 78 NHL patients and 122 healthy individuals by PCR-RFLP technique. The presence of the *VEGF 936T* allele was found to significantly associate with worse prognosis of the disease (expressed by the highest International Prognostic Index (IPI)) (0.41 versus 0.20, *P* = 0.044 for IPI 4 among patients having and lacking the *T* allele). The *VEGF 936T* variant was also more frequent among patients with IPI 4 than in controls (OR = 3.37, *P* = 0.029). The *bFGF −921G* variant was more frequently detected among patients with aggressive as compared to those with indolent histological subtype (0.37 versus 0.18, *P* = 0.095) and healthy individuals (0.37 versus 0.19, OR = 2.51, *P* = 0.038). These results imply that VEGF and bFGF gene polymorphisms have prognostic significance in patients with NHL.

## 1. Introduction

Non-Hodgkin's lymphomas (NHLs) form a heterogeneous group of lymphoproliferative neoplasms with different presenting features, clinical course, and response to treatment. NHLs constitute approximately 3% of all malignancies. According to geographic location, the incidence of lymphomas varies from 2 to 18 cases per 100,000 people per year. The course of disease depends on various biological and clinical parameters as well as therapeutic decisions at the beginning of treatment. Biological mechanisms leading to the development of NHL are not clearly understood.

Lymphoma growth and progression may be enhanced by angiogenesis. Both vascular endothelial growth factor (VEGF) and basic fibroblast growth factor (bFGF) play an important role in this process. VEGF is a potent mediator of angiogenesis by autocrine stimulation of tumour cells as well as paracrine influences of the proangiogenic tumour microenvironment [1]. There are some reports suggesting that VEGF expression in lymphomas reflects their proliferative activity. Increased serum level of VEGF has been reported in aggressive lymphoma, whereas VEGF expression in indolent lymphomas is low. The prognostic and predictive value (as a possible treatment target) of increased microvessel density and angiogenic factors in lymphomas is still controversial due to the heterogeneity of diseases, different classifications, and methods of analysis (immunohistochemistry, serum levels of angiogenic markers, mRNA extraction, etc.). In B-cell lymphomas, VEGF protein and mRNA have been identified in diffuse large B-cell lymphoma (DLBCL), mantle cell lymphoma (MCL), central nervous system DLBCL, and viral related lymphomas [2]. A large study of 200 patients showed that high pretreatment levels of both serum VEGF and bFGF were independent prognostic factors for survival in multivariate analysis [3].

The human *VEGF* gene is located on chromosome 6p21.3 and is organized into 8 exons separated by 7 introns [4]. Several single nucleotide polymorphisms (SNPs) have been described in the *VEGF* gene that are related to VEGF protein production, including three promoter region SNPs −2578 C/A (rs699947) [5], −1154 G/A (rs1570360) [5, 6], −460 C/T (rs833061) [7–9], one 5′-untranslated region (5′ UTR) SNP +405 G/C (rs2010963) [7–9], and one 3′-untranslated region (3′ UTR) SNP +936 C/T (rs3025039) [10].

The present study focused on the latter polymorphism, C+936T in the 3′ UTR [10]. VEGF plasma levels were reported to be significantly lower in carriers of the *936T* allele what could be attributed to the 936 C/T exchange leading to the loss of a potential binding site for transcription factor AP-4 (activating enhancer binding protein 4). 

The human *bFGF* gene is located on chromosome 4 [11]. Polymorphisms within the promoter region of the *bFGF* gene may interfere with existing transcription factor binding sites or produce new binding sites and, therefore, influence *bFGF* gene expression [12]. 

In the present study C to T substitution at position 936 within the 3′-untranslated region of the *VEGF* gene and C to G substitution at position −921 within the promoter region of the *bFGF* gene were analysed in order to determine whether the presence of these allelic variants is associated with susceptibility and progression of the disease in NHL patients. 

## 2. Materials and Methods

### 2.1. Patients and Controls

The present study was conducted on a group of 78 consecutive patients (47 males and 31 females; aged 19–85 years, average age 51 years) recruited at the Department of Haematology, Wroclaw Medical University. The study was conducted in accordance with the Helsinki Declaration of Human Rights, and all the participants gave informed consent. For patients' characteristics, see Table 1. In addition, 122 healthy individuals (volunteer blood/bone marrow donors) of both sexes served as controls.

### 2.2. VEGF and bFGF Genotyping

DNA was isolated from the whole blood taken on EDTA using the Qiagen DNA Isolation Kit (Qiagen GmbH, Hilden, Germany). The *VEGF* and *bFGF* alleles were detected using a polymerase chain reaction restriction fragment length polymorphism (PCR-RFLP) technique. 

In brief, DNA was extracted from peripheral blood taken on EDTA using silica membranes (QiAmp Blood Kit, Qiagen, Hilden, Germany) following the recommendations of the manufacturer. A 208 bp long fragment of the 3′ UTR of the *VEGF* gene (rs3025039) was amplified using the following primers as previously described [13]: forward, 5′-AAG GAA GAG GAG ACT CTG CGC AGA GC-3′, reverse, 5′-TAA ATG TAT GTA TGT GGG TGG GTG TGT CTA CAG G-3′. The following primer pair was used for amplification of a 437 bp long fragment of the *bFGF* gene promoter region (rs308395): 5′-TGA GTT ATC CGA TGT CTG AAA TG-3′ and 5′-TAAC TTG AAT TAG ACG ACG CAG A-3′ [12]. The PCR cycling conditions were as follows: 94°C for 3 min; followed by 30 cycles of 94°C for 30 s; 60°C for 30 s; 72°C for 30 s; with a final elongation step at 72°C for 7 min. The PCR products were analysed by electrophoresis in a 2% agarose gel with ethidium bromide and visualized under UV. Then the PCR products specific for the *VEGF* and *bFGF* genes were digested with *Nla*III and *Bse*NI restriction endonucleases (New England BioLabs Inc.), respectively, and analysed on an agarose gel. Electrophoresis demonstrated the original 208 bp fragment (individuals homozygous for the *VEGF C* allele, lacking the *Nla*III site), three fragments of 208, 122 and 86 bp in length (heterozygous individuals), or two fragments of 122 and 88 bp (individuals homozygous for the *VEGF T* variant). For *bFGF* polymorphism, the following electrophoresis patterns were observed: the original 437 bp fragment (homozygous individuals for the *bFGF C* allele, lacking the *Bse*NI site), three fragments of 437, 370 and, 67 bp in length (heterozygous individuals), or two fragments of 370 and 67 bp (individuals homozygous for the *bFGF G* variant).

### 2.3. Statistical Analysis

Statistical evaluation was performed using Statistica 5.5 for Windows software. Genotype and allele frequencies were compared between the study groups by Fisher's exact or Chi-square test with Yates' correction as appropriate. The odd's ratio (OR) was calculated by Haldane's modification of Woolf's method, and the significance of its deviation from unity was estimated by Fisher's exact test. Survival analyses were performed employing Kaplan-Meier analysis and log rank test. Probability values <0.05 were considered statistically significant, and those between 0.05 and 0.1 were considered as indicative of a trend.

## 3. Results

### 3.1. Distribution of *VEGF* and *bFGF* Alleles and Genotypes

Distribution of *VEGF* (rs3025039, 936 C>T) and *bFGF* (rs308395, −921 C>G) genotypes was analysed in NHL patients and compared with healthy individuals (Table 2). 

None of the patients presented with *VEGF* or *bFGF* homozygous mutated genotype. The *VEGF CC*, *CT*, and *TT* genotypes were respectively detected in 61 (78%), 17 (22%) and none of the patients and in 101 (83%), 13 (11%) and 8 (6%) controls. The *bFGF CC* and *CG* genotypes were detected in 57 (73%) and 21 (27%) patients and in 99 (81%) and 22 (18%) controls, respectively. The *GG* homozygosity was very rare. Only one healthy individual was carrying this genotype. 

The allelic frequencies were as follows: *VEGF* for patients—*C*: 0.891, *T*: 0.109; and controls *C*: 0.881, *T*: 0.119; and *bFGF* for patients—*C*: 0.865, *G*: 0.135; and controls—*C*: 0.902, *G*: 0.098. 

The comparison of *VEGF* and *bFGF* genotypes and allele frequencies in our group of healthy subjects with the data coming from other studies did not show significant differences. Similar results were obtained in other European populations (i.e., those reported by Beránek et al. [12], Galimberti et al. [14], Kariž et al. [15], and Petrovič et al. [16]).

The presence of the *bFGF G* variant was slightly more frequent among patients (27%) than controls (19%) (OR = 1.61, *P* = 0.24 (Table 3)). This difference reached statistical significance for a subgroup of aggressive NHL patients. Among 38 patients with aggressive NHL, 14 (37%) were carrying the *bFGF G* variant as compared to 23 out of 122 (19%) healthy individuals (OR = 2.51, *P* = 0.038) (Table 3).

### 3.2. Analysis of Association of the *VEGF* Polymorphic Features with the Course of NHL

Although no significant association between *VEGF* polymorphism and susceptibility to NHL was found, a correlation between the *VEGF CT* genotype (presence of the *T* variant) and the course/progression of the disease could be identified. Patients characterized by IPI 3 and/or 4 were more frequently carrying the *VEGF T* allele. A strong tendency was observed towards the higher frequency of the *VEGF T* allele among patients with intermediate high/high IPI (3 or 4) as compared to patients with low or intermediate IPI (1 or 2) (*P* = 0.077) and controls (OR = 2.22, *P* = 0.093) (Table 3).

A significant association was observed when patients carrying the *T* variant with IPI 4 were compared to those with lower IPI (7/17 versus 10/61, *P* = 0.044) and healthy individuals (7/17 versus 21/122, OR = 3.37, *P* = 0.029) (Table 3). Analyses of the distribution of *VEGF* genotypes with respect to the stage, presence of B symptoms, or aggressiveness of the disease did not show any significant correlation. 

No association was also found between the polymorphism studied and response to treatment. There was no significant difference in the distribution of the *VEGF* alleles among patients in complete or partial remission or in those who did not respond to treatment.

A slight tendency was observed towards less favourable overall survival among NHL patients with the *VEGF T* variant. Thirty-one out of 61 (51%) patients carrying the *CC* wild type genotype as compared to 12 out of 17 (71%) patients carrying the *CT* genotype died in the course of the disease (*P* = 0.24). This difference reached statistical significance when only the patients with indolent disease were considered. In this group of patients, fatal cases constituted 48% (15/31) and 89% (8/9) of wild type and mutated genotype carriers (*P* = 0.05), respectively.

### 3.3. Relationships of the *bFGF* Polymorphic Variants with Progression of NHL

To assess whether *bFGF* genotypes are associated with unfavourable progression of NHL, distributions of *bFGF* alleles and genotypes were compared among patients with different clinical characteristics and IPI. It was found that aggressive NHL patients more than twice as frequently presented with the *bFGF G* variant as those with the indolent histological type. Among 38 patients with aggressive NHL, 14 (37%) were carrying the *T* variant as compared to 7 out of 40 patients (18%) with indolent disease (*P* = 0.095) and to 23 out of 122 (19%) healthy individuals (OR = 2.51, *P* = 0.038) (Table 3). No other relationships were observed.

## 4. Discussion

The presence of the polymorphic variants located within the genes coding for two proangiogenic factors (VEGF and bFGF) were found to be associated with susceptibility and progression of the disease in NHL patients. The presence of the *VEGF 936T* allele was found to significantly associate with worse prognosis while the *bFGF  −921G* variant was more frequently detected among NHL patients with aggressive histological subtype of the disease.

There is an emerging evidence suggesting that tumour progression of haematological malignancies depends on the induction of new blood vessel formation [17, 18]. The most important proangiogenic agent is VEGF acting as a potent mediator of angiogenesis by autocrine and paracrine activity affecting proliferation and survival of leukaemia/lymphoma cells in addition to tumour vascularisation. Also bFGF plays an important role in vascular responses by increasing endothelial cell proliferation, stimulating migration, and promoting angiogenesis [19]. Therefore analyses of VEGF and bFGF expressions in haematological malignancies, including NHL, attracted the attention of many researchers [3, 20–22], while there have been fewer studies addressing the role of polymorphic features located within the genes coding for these two angiogenic factors [14, 23, 24] with only two reports describing the association of *VEGF* genetic polymorphisms with the clinical characteristics of NHL in Asian patients from China [23] and Korea [24].

Also our former study showed elevated VEGF serum levels in patients with B-cell NHL as compared with the control group of healthy people [21, 25]. Moreover, in the NHL group VEGF serum level was correlated with IPI risk factors, but the levels of VEGF were not significantly different in aggressive or indolent NHL patients. The present study documents the association between the polymorphic features located within the *VEGF* and *bFGF* genes in NHL patients with prognosis and progression of the disease. We found that patients carrying the *VEGF T* variant more frequently presented with a higher IPI score. This is a novel, observation, not previously described. The observed relationship is quite unexpected because the association of the *VEGF T* variant with lower *VEGF* gene expression and cytokine production [10], and the higher pre-treatment serum levels with unfavourable course of the disease [3, 21] were reported. On the other hand, the value of plasma or serum VEGF levels as a prognostic marker in lymphoma has been inconsistent. Recently, in the SWOG study (Southwest Cancer Chemotherapy Study, formerly the Southwest Oncology Group) there was no correlation between urine and serum VEGF levels and IPI [26].

In the present study we did not analyse either *VEGF* or *bFGF* gene expressions (serum levels) in our cohort of Polish patients with NHL. Therefore we cannot decisively conclude that the observed association of the *VEGF *variant with IPI is not associated with expression of the *VEGF* gene. This should be analysed in a separate study involving a larger cohort of patients and healthy controls.

In this study we also detected a previously unreported association between the *bFGF* gene polymorphism and a more unfavourable course of the disease. The frequency of the *bFGF G* variant was higher in patients with aggressive NHL as compared with patients with indolent forms of the disease and controls. According to our knowledge, this is the first study describing the association of the *bFGF *gene polymorphism with NHL. The results published so far have considered the effect of the relationships of polymorphisms in the promoter region of the basic fibroblast growth factor gene with proliferative diabetic retinopathy [16] or myocardial infarction [15] in patients with type 2 diabetes. Moreover, the *bFGF* (rs308395, −921 G>C) gene polymorphism studied was described with respect to the its effect on mRNA expression or protein production. Thus it is difficult to speculate on the association between this polymorphism, bFGF expression, and disease susceptibility or progression in NHL patients investigated in the present study. It seems that the *bFGF G* allele might be associated with a higher bFGF serum level in NHL patients. However, this relationship warrants further study. As for bFGF expression, Slaven et al. [3] reported elevated serum bFGF levels in patients with NHL. However, they did not observe significant differences in the bFGF levels between patients who presented with the different histological subtypes.

Due to the lack of the data on the VEGF and bFGF serum levels in patients and controls investigated in the present study, we were not able to analyse the relationship between the polymorphism and expression of the genes in both groups of individuals tested, to compare these results in patients and controls, and to try to detect new and confirm the previously published results on association between serum levels and development of the disease. This constitutes a limitation of our study. 

However, the information from the present study contributes to other data on genetic factors associated with NHL [27, 28]. Obviously, due to the quite small number of individuals investigated, our findings need to be confirmed by extended studies involving an independent cohort of patients before drawing the final, general conclusion regarding the association of *bFGF *gene polymorphisms with NHL. It would also be of interest to relate *bFGF* gene polymorphisms and *bFGF* gene expression/cytokine production in NHL patients with different manifestations of the disease. These warrant further studies.

## Figures and Tables

**Table 1:** Patients characteristics.

Characteristics	NHL patients (*n* = 78)
Sex	
Female	31
Male	47
Age	
<60 yrs	51
>60 yrs	27
Ann Arbor stage	
I/II	13
III/IV	65
B symptoms	
Absent	13
Present	65
Serum LDH	
Normal	49
Elevated*	29
Performance status (ECOG)	
<2	24
≥2	54
Number of extranodal sites	
<2	55
≥2	23
Serum *β*2-microglobulin	
Normal	20
Elevated^†^	35
Unknown	23
IPI risk groups	
Low/intermediate low (1, 2)	40
Intermediate high/high (3, 4)	38
Histological aggressiveness	
Indolent	40
Follicular	17
Small lymphocytic	15
Other B cells	8
Aggressive	38
Diffuse large B-cell lymphoma	28
Mantle	5
Lymphoblastic	2
Peripheral T cell	3
Survival	
Dead	47
Alive	31
Response to treatment	
Complete remission	35
Partial remission	30
No response	13

NHL: non-Hodgkin's lymphoma; LDH: serum lactate dehydrogenase; ECOG: Eastern Cooperative Oncology Group; IPI: International Prognostic Index.

*>480 U/L; ^†^>1.80 mg/L.

**Table 2:** Distribution of the *VEGF *and *bFGF* genotypes in patients with non-Hodgkin's lymphoma (NHL) and healthy individuals.

Polymorphism	NHL patients *n* (%)	Controls *n* (%)
*VEGF* (pp. 936 C>T)		
CC	61 (78%)	101 (83%)
CT	17 (22%)	13 (11%)
TT	0 (0%)	8 (6%)
*bFGF* (pp. −921 C>G)		
CC	57 (73%)	99 (81%)
CG	21 (27%)	22 (18%)
GG	0 (0%)	1 (1%)

**Table 3:** Patients characteristics with respect to the detected associations with the *VEGF T* and *bFGF G* variants in comparison with healthy population. The *bFGF G* variant was more frequently detected among patients with aggressive histological subtype of NHL. The *VEGF T* variant was more frequently detected among patients presented with high IPI. Individuals carrying the *VEGF T* variant are over three times more likely to develop NHL characterized by the highest IPI score, while those with the *bFGF G* allele are over twice more likely to present with aggressive histological type of the disease.

NHL patients	*N*	*VEGF pp. 936 *		*bFGF pp. −921 *	
		*CC *	*T *	*CC *	*G *
		*n* (%)	*n* (%)	*n* (%)	*n* (%)
IPI risk groups					
Low/intermediate low (1, 2)	40	35 (87.5%)	5^§^ (12.5%)	31 (77.5%)	9 (22.5%)
Intermediate high/high (3, 4)	38	26 (68.4%)	12^§,¶^ (31.6%)	26 (68.4%)	12 (31.6%)
Low/intermediate (1–3)	61	51 (83.6%)	10^#^ (16.4%)	46 (75.4%)	15 (24.6%)
High (4)	17	10 (59%)	7^#,∗∗^ (41%)	11 (64.7%)	6 (35.3%)

Histological aggressiveness					
Indolent	40	31 (77.5%)	9 (22.5%)	33 (82.5%)	7^†^ (17.7%)
Aggressive	38	30 (78.9%)	8 (21.1%)	24 (63.2%)	14^†,‡^ (36.8%)
Patients	78	61 (78.2%)	17 (21.8%)	57 (73%)	21* (27%)
Healthy individuals	122	101 (82.8%)	21^¶,∗∗^ (17.2%)	99 (81.1%)	23^∗,‡^ (18.9%)

NHL: non-Hodgkin's lymphoma; IPI: International Prognostic Index; *(NHL patients versus controls) OR = 1.61, *P* = 0.24; ^†^(patients with aggressive versus patients with indolent disease) *P* = 0.095; ^‡^(patients with aggressive disease versus controls) OR = 2.51, *P* = 0.038; ^§^(patients with intermediate high/high IPI (3, 4) versus low/intermediate low IPI (1, 2)) *P* = 0.077; ^¶^(patients with intermediate high/high IPI (3, 4) versus controls) OR = 2.22, *P* = 0.093; ^#^(patients with high (4) versus low/intermediate (1–3) IPI) *P* = 0.044; **(patients with high IPI (4) versus controls) OR = 3.37, *P* = 0.029.
